# Enhanced mechanisms of pooling and channel attention for deep learning feature maps

**DOI:** 10.7717/peerj-cs.1161

**Published:** 2022-11-21

**Authors:** Hengyi Li, Xuebin Yue, Lin Meng

**Affiliations:** 1 Graduate School of Science and Engineering, Ritsumeikan University, Kusatsu, Shiga, Japan; 2 College of Science and Engineering, Ritsumeikan University, Kusatsu, Shiga, Japan

**Keywords:** DNNs, Max pooling, Average pooling, FMAPooling, Self-attention, FMAttn

## Abstract

The pooling function is vital for deep neural networks (DNNs). The operation is to generalize the representation of feature maps and progressively cut down the spatial size of feature maps to optimize the computing consumption of the network. Furthermore, the function is also the basis for the computer vision attention mechanism. However, as a matter of fact, pooling is a down-sampling operation, which makes the feature-map representation approximately to small translations with the summary statistic of adjacent pixels. As a result, the function inevitably leads to information loss more or less. In this article, we propose a fused max-average pooling (FMAPooling) operation as well as an improved channel attention mechanism (FMAttn) by utilizing the two pooling functions to enhance the feature representation for DNNs. Basically, the methods are to enhance multiple-level features extracted by max pooling and average pooling respectively. The effectiveness of the proposals is verified with VGG, ResNet, and MobileNetV2 architectures on CIFAR10/100 and ImageNet100. According to the experimental results, the FMAPooling brings up to 1.63% accuracy improvement compared with the baseline model; the FMAttn achieves up to 2.21% accuracy improvement compared with the previous channel attention mechanism. Furthermore, the proposals are extensible and could be embedded into various DNN models easily, or take the place of certain structures of DNNs. The computation burden introduced by the proposals is negligible.

## Introduction

Deep neural networks (DNNs) have achieved great success in various domains, including object detection (Yue et al., 2022b; Dong et al., 2022), natural language processing, human health care (Saho et al., 2022; Chen et al., 2020), cultural heritage protection (Yue et al., 2022a; Fujikawa et al., 2022), and intelligent control (Li et al., 2020; Liu, Yu & Cang, 2018, 2019; Liu et al., 2020), *etc.* However, the convolution operation extracts specific data rather than generalized data, which are sensitive to the location of the input feature maps. As a result, this leads to serious overfitting (Li et al., 2021). As for the pooling function, it provides an effective solution by generalizing the presence of the input features. In addition, it also reduces the calculation consumption for the networks. Thus, the pooling functions have been widely adopted in DNNs. For example, VGGNet employs five max pooling layers (Liu & Deng, 2015), GoogLeNet takes four max-pooling layers (Szegedy et al., 2015), ResNet and DenseNet both adopt one max pooling layer after the first convolutional layer (He et al., 2016; Huang et al., 2017), and all of the DNN architectures have an adaptive average pooling layer before the last classification layer/block.

The pooling function includes a series of methods, including max pooling operation (Zhou & Chellappa, 1988), average of a rectangular neighborhood, L2 norm of adjacent neighborhoods, weighted average based on the distance from the central unit, and so on. The characteristics of pooling can be summarized into two points. First is the feature invariant of the function. The pooling operation samples the inputs by making the representation of the feature maps approximately invariant to small size with the summary statistic of nearby features. Ian, Yoshua & Aaron (2016) determined that for features, invariance to local translation can be a useful property when it is more crucial for whether they are, rather than exactly where they are. Second, the down-sample function reduces the redundant information with key features remaining. As a result, the complexity of the network, the floating-point operations (FLOPs), and the memory consumption are reduced. Further, the effect of the over-fitting problem is alleviated and the generalization ability of the network is improved. As a result, pooling has been a vital function for CNNs. However, regardless of the benefits of the pooling, the down-sampling of the function results in information loss more or less inevitably.

Based on the max pooling and average pooling, which are the most widely adopted functions in DNNs, the research makes contributions as follows: we propose the FMAPooling pooling function and the improved channel attention mechanism FMAttn to enhance the representation ability of feature maps. And the effectiveness of the proposals are verified with sufficient experiments. Furthermore, the proposals could be easily integrated into various DNN architectures by adding directly or replacing certain structures of DNNs.

## Related work

This section introduces the research concerned with pooling functions. Max pooling and average pooling functions are the basic pooling computations, which derive multiple pooling strategies as follows.

Global average pooling was proposed to take over the traditional fully connected (FC) layers for classification in CNNs (Lin, Chen & Yan, 2014). The function features in adopting the overall spatial averages of each feature map as the confidence of categories and then passing the vector into the last softmax layer for classification.

In general, the input size of CNNs is fixed while the scale of original data varies greatly. As a result, the data need to be unified to a fixed size artificially, which may lead to information loss for large-size images. Spatial pyramid pooling (SPP) was designed to eliminate the problem by generating the fixed-length representation for arbitrary size/scale inputs at the top of the last convolutional layer (He et al., 2015). Then, the representation length for the next classification layer is unified.

Center pooling takes the summary of the maximum values in both the horizontal and vertical directions. This contributes to the detection of center key points for object detection (Duan et al., 2019).

Corner pooling helps to better localize the corners for the object detection (Law & Deng, 2020). To detail the method, assuming that it is needed to recognize if pixel A is the top-left corner of the object: for each channel of each feature map, it adopts the summary of the maximum values in two directions as the final pixels, *i.e*., the horizontal and vertical directions.

In summary, all of the pooling functions are based on the max pooling and/or average pooling, and the difference lies in the operation objects. Then, in this article, we focus on the max pooling and average pooling functions, which are $k \times k$ sliding window objects, to improve the expressibility of DNNs. This is also the most widely adopted pooling function.

In terms of the attention methods for DNNs, Vaswani et al. (2017) proposed the first attention mechanism, the Transformer, and achieved SOT results of the time on two translations. Since then, multiple attention mechanisms for DNNs are proposed. For vision tasks, the attention mechanism is mainly to adaptively exploit the inter-relationship of features referring to different dimensions including channel and spatial, and different scopes including local and global.

Hu, Shen & Sun (2018) proposed the “Squeeze-and-Excitation” (SE) block to highlight the informative features while suppressing the redundant ones at the channel level. The method utilizes the squeeze and excitation operations to dynamically recalibrate the channel interdependencies. The ILSVRC2017 classification submission based on the channel attention mechanism won the champion.

Wang et al. (2020) proposed an efficient channel attention (ECA) mechanism. The technique acquires the channel attention by applying a fast 1*D* convolution with the kernel size being adaptively determined by a non-linear mapping of the channel dimension. Thus the local cross-channel interaction is realized. In addition, the proposal is extremely lightweight.

Woo et al. (2018) proposed the Convolutional Block Attention Module (CBAM) for convolutional neural networks. The method adopts both channel and spatial level attention for feature refinement. The channel attention utilizes max pooling and average pooling to produce finer channel attention weights. The spatial attention exploits the inter-spatial interdependencies of feature elements by applying the max pooling and average pooling along the channel axis and then concatenates the features to generate the spatial feature descriptor. The mechanism achieves the best performances for various state-of-the-art DNN models on ImageNet1K, MS COO, and VOC 2007. The three self-attention proposals are the current most widely applied methods. It is worth noting that attention mechanisms are all based on pooling functions. By revisiting and evaluating the three methods, we propose a novel channel attention mechanism by utilizing both max pooling and average pooling functions.

## Method

This section provides the theoretical basis of the proposals.

### FMAPooling

The proposal FMAPooling is introduced in this section. At first, the both pooling functions are deeply studied.

Max pooling is the most widely applied approach. The function takes the maximum pixel value of the batch selected, *i.e*., a group of pixels determined by the max-pooling kernel size. It recognizes and retains the most prominent characteristics of the feature maps. Average pooling takes the average pixel value of the batch selected, which results in smoothing out the feature maps. The mathematical formulas for the two pooling functions are as follows:

(1) $${P_{Max}} = Max_{i,j = 0,0}^{i,j = h,w}\left\{ {{x_{i,j = 0,0}},{x_{i,j = 0,1}},{x_{i,j = 1,0}},...,{x_{i,j = h,w}}} \right\}$$

(2) $${P_{Avg}} = \displaystyle{1 \over {h \times w}}\sum\limits_{i,j = 0,0}^{h,w} {{x_{i,j}}}$$

where ${P_{Max}}$ and ${P_{Avg}}$ denote the max pooling and average pooling operation on a single group of data in the feature maps determined by the pooling kernel; $h \times w$ denotes the kernel size for pooling operation, which is up to the case. In general, the kernel size is less than the size of input feature maps $H \times W$ and is set to $2 \times 2$; $i \in [0,h]$ and $j \in [0,w]$; $Max\left\{ * \right\}$ denotes the max value of the data group $\left\{ {{x_{i,j = 0,0}},{x_{i,j = 0,1}},{x_{i,j = 1,0}},...,{x_{i,j = h,w}}} \right\}$; ${x_{i,j}}$ denotes the pixel element of the ${i_{th}}$ column and the ${j_{th}}$ row determined by the pooling kernel $h \times w$; Furthermore, ${P_{Max}}\left\{ * \right\}$ and ${P_{Avg}}\left\{ * \right\}$ denote the pooling operation on the whole input feature maps.

**Figure 1:**
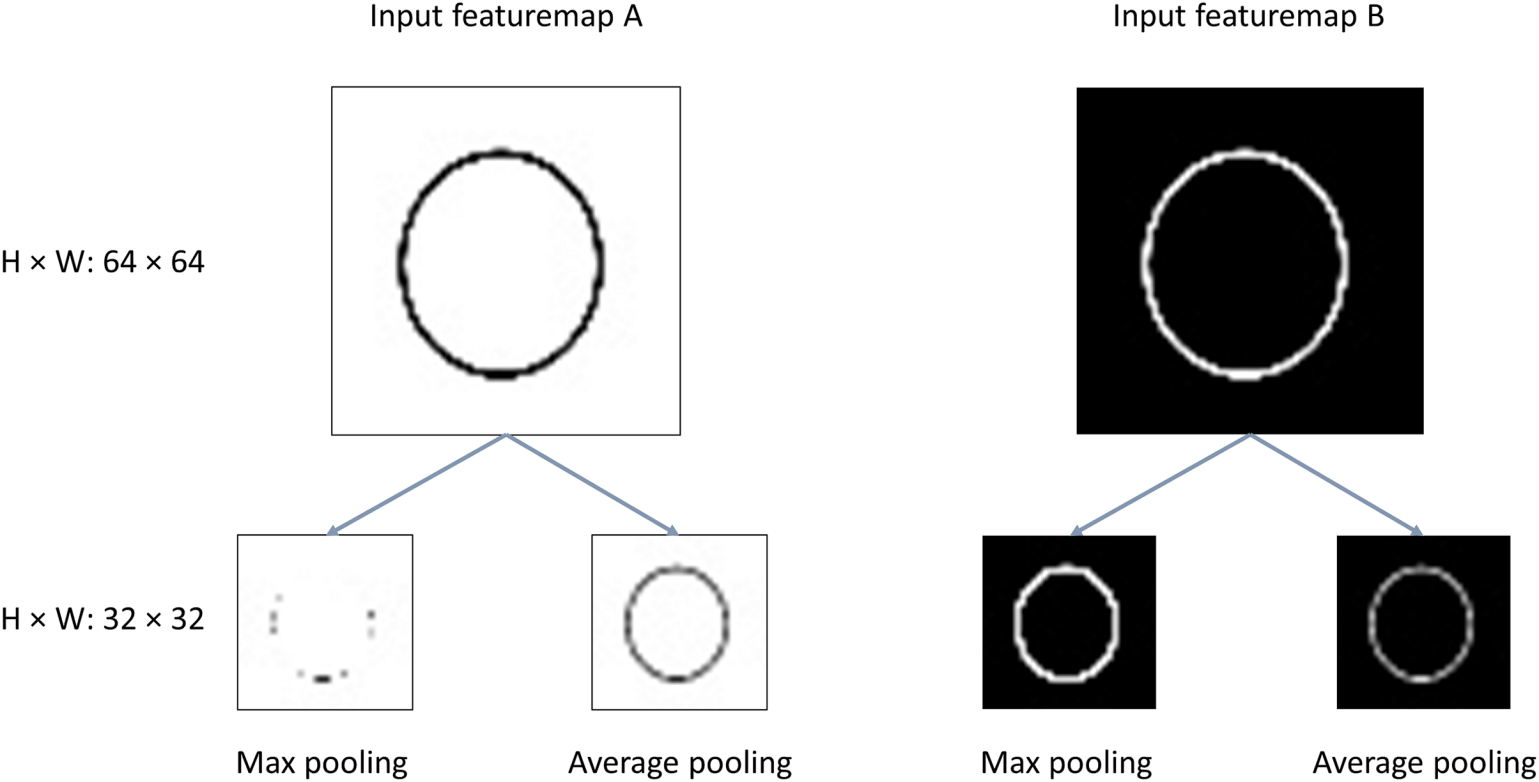
Illustration of max/average pooling. H, Height; W, width.

It is hard to say which pooling performs better than another. Generally, it is up to the exact tasks (Yu et al., 2014). For clarity, we made an experiment to provide a quite intuitive and concise illustration of the both pooling functions extracting features from two different inputs, as is shown in Fig. 1. The task is to recognize the circle in the images. With the pooling operation:

- Cases with inputs like feature map A: the informative features vanish greatly for the output of max pooling; And the average pooling retains the most of the valuable features;
- Cases with inputs like feature map B: it is obvious the max pooling outperforms average pooling with more clearer features extracted from the original images.

Conclusions could be drawn that the max pooling extracts features better for images in which the background of the images is dark and the features located on the lighter pixels of the images, like MNIST dataset which corresponds to the case of input feature map B. However, for the cases of input like feature map with the background of the images is white the features located on the darker pixels, max pooling is impotent and the average pooling performs better. Actually, the tasks of DNNs are quite complicated rather than black-white or gray images. For example, the datasets like ImageNet, CIFAR10/100, *etc*, the informational features need to be extracted from rather complex images. Then, a single max pooling or average pooling inevitably damages the useful features in the images. Thus, we propose the fused max-average pooling (FMAPooling) functions to enhance the representations of the feature maps after being down-sampled by pooling operation.

**Figure 2:**
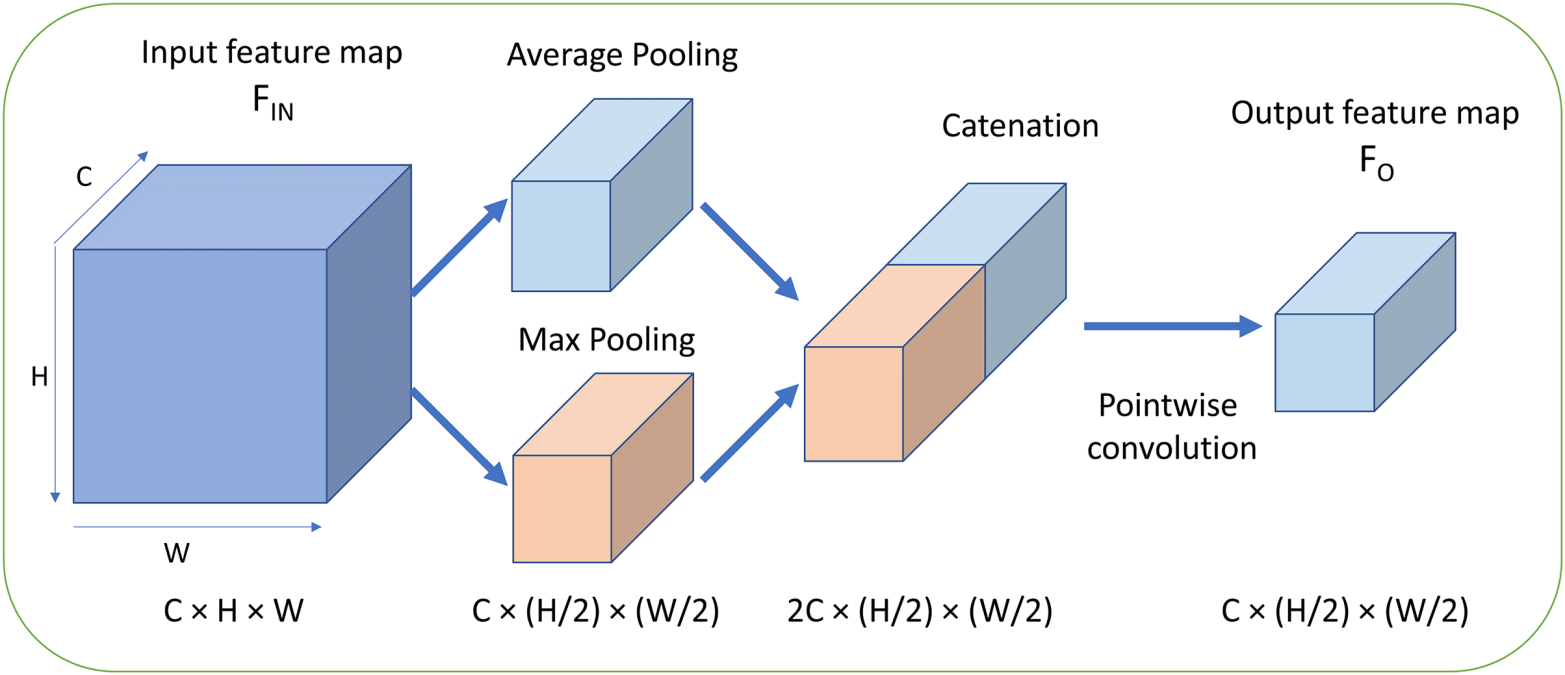
Fused max-average pooling (FMAPooling). C, Channels; H, height; W, width.

The structure of the proposal FMAPooling is shown in Fig. 2:

The mathematical function of FMAttn is as follows.

(3) $${F_O} = Con{v_{pw}}\left( {{F_{cat}}\left( {{P_{Max}}\left\{ {{F_{IN}}} \right\},{P_{Avg}}\left\{ {{F_{IN}}} \right\}} \right)} \right)$$

where $Con{v_{pw}}$ denotes the point-wise convolution; ${F_{cat}}$ denotes the concatenation operation; ${F_{IN}}$ and ${F_O}$ denote the input and output feature maps of FMAttn.

The FMAttn consists of three steps. First, the input feature maps are processed with max pooling and average pooling respectively, with the parameters *h* and *w* of function ${P_{Max}}$ and ${P_{Avg}}$ both being set to 2 in general, which is less than the size $H \times W$ of input feature maps. Second, the two pooling outputs are concatenated together resulting in channels doubled. Third, the concatenated feature maps are convoluted with pointwise convolution to fuse the both features and reduce the doubled dimensionality by half as well as the introduced computation overhead by concatenation at the same time. In addition, the pointwise convolution is also followed by a batch normalization (BN) layer and a ReLU layer. Then, with the concatenation and the pointwise convolution operations, the both pooling features are merged and then the features are enhanced.

### FMAttn

**Figure 3:**
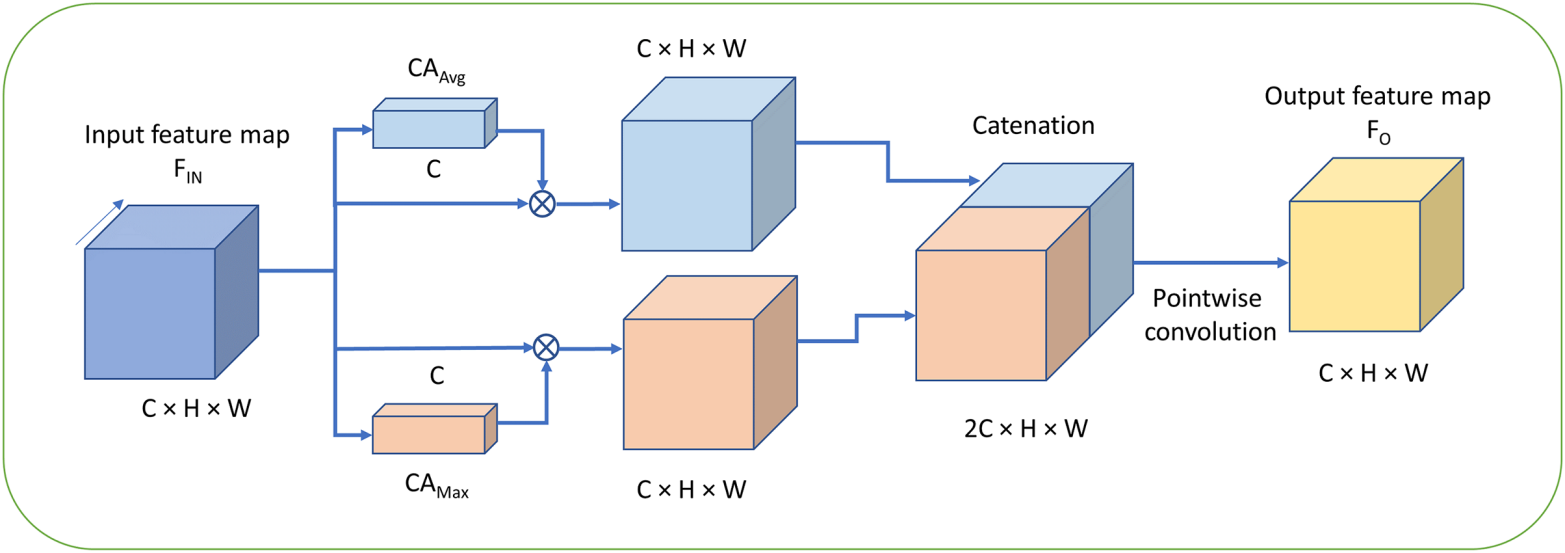
Fused max-average attention (FMAttn). C, Channels; H, height; W, width.

For the channel attention mechanism, Woo et al. (2018) have proposed the convolutional block attention module (CBAM) which adopts the channel attention method CAM by utilizing both the max pooling and average pooling in the stage of channel attention. However, the CAM processes the two outputs generated by the two pooling functions with the shared MLP respectively at first. Second, the both pooling features are simply added together which is then denoted as MP_features. Finally, the original feature maps are multiplied by MP_features and then the channel-attention feature maps are obtained. The method CAM achieves better performance compared with the SENet and ECANet. Furthermore, we propose a novel mechanism for channel attention which is defined as FMAttn by utilizing the max pooling and average pooling. The structure of FMAttn is detailed in Fig. 3.

The mathematical function of FMAttn is as follows.

(4) $$C{A_{Max}} = S\left( {MLP\left( {{P_{Max}}\left\{ {{F_{IN}}} \right\}} \right)} \right)$$

(5) $$C{A_{Avg}} = S\left( {MLP\left( {{P_{Avg}}\left\{ {{F_{IN}}} \right\}} \right)} \right)$$

(6) $${F_O} = Con{v_{pw}}\left( {{F_{cat}}\left( {C{A_{Max}} \otimes {F_{IN}},C{A_{Avg}} \otimes {F_{IN}}} \right)} \right)$$

where *S* denotes the sigmoid function; *h* and *w*, the parameters of ${P_{Max}}$ and ${P_{Avg}}$ which are presented in Eqs. (1) and (2), equal the feature map size H and W respectively in Eqs. (4) and (5); *MLP* denotes the multi-layer perceptron with two convolutional layers followed by Hardtanh function respectively; $C{A_{Max}}$ denotes the channel attention with max pooling, and the generated features by are denoted as $C{A_{Max}}:\left\{ {{m_1},{m_2},{m_3},...,{m_k}} \right\}$; $C{A_{Avg}}$ denotes the channel attention with average pooling, and the generated features are denoted as $C{A_{Avg}}:\left\{ {{a_1},{a_2},{a_3},...,{a_k}} \right\}$; *k* equals the channels *C* of input feature maps; ${F_{cat}}$ denotes the concatenation operation; $\otimes$ denotes the convolution which is pointwise and depthwise in this study; $Con{v_{pw}}$ denotes the point-wise convolution which reduce the dimensionality by half; And ${F_O}$ denotes the output feature map of the proposal FMAttn.

**Figure 4:**
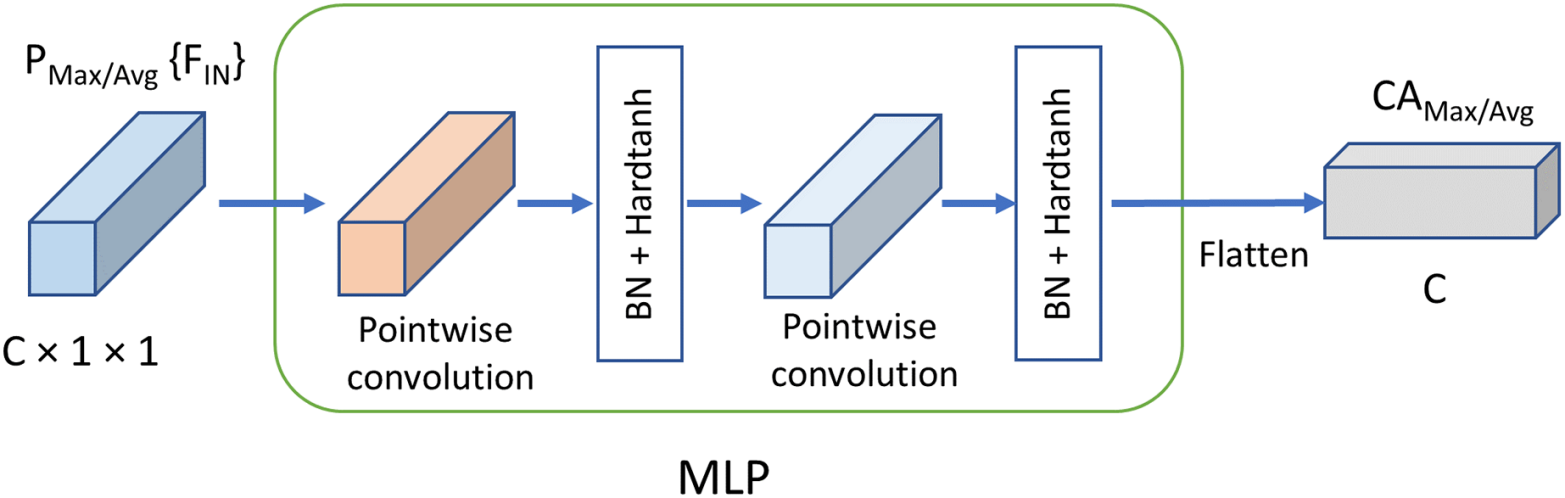
Mechanism of MLPC. C, Channels; BN, batch normalization.

Differing from the excitation structure adopted in SE block, the feature dimensions for *MLP* remains the same as the original input without reducing the dimensions, as is shown in Fig. 4. The channel weights based on max pooling channel attention and average pooling channel attention, *i.e*., $C{A_{Max}}$ and $C{A_{Avg}}$, are generated respectively at first. And then, $C{A_{Max}}$ and $C{A_{Avg}}$ convolve with the input feature maps respectively to generate the self-attention feature maps. Finally, the two groups of feature maps are concatenated which is followed by the pointwise convolution to fuse and enhance the feature map representations further. The mechanism is basically different from the channel attention proposed in previous methods and has better representation properties.

## Experiments

In this section, a series of experiments are conducted to demonstrate the effectiveness of the proposals FMAPooling and FMAttn. In detail, VGG, ResNet, and MobileNetV2 are adopted to make experiments on datasets CIFAR10/100 and ImageNet100 respectively.

The DNN networks adopted in the experiments belong to the most representative and significant DNN architectures. VGG architecture is proposed in Simonyan & Zisserman (2015) as a milestone for the development of DNNs and is still one of the most preferred choices for its excellent performance in feature extraction. ResNet is proposed in He et al. (2016) as one of the greatest breakthroughs which eliminates both the degradation and saturation problems of DNNs. Both the two DNN architectures are widely referenced and adopted by the following studies Zhang & Schaeffer (2019), Zhang et al. (2018). MobileNetV2 features in inverted residual structure with linear bottlenecks, which is a specially designed lightweight DNN architecture for mobile devices (Howard et al., 2017). In particular, VGG architecture adopts max pooling after each group of convolutional layers, while ResNet takes only one max pooling layer after the first convolutional layer and MobileNetV2 has no pooling function. As a result, FMAPooling is just applied to VGG to test the performance of the method. In terms of the FMAttn, experiments are conducted on all the three DNN architectures to verify the effectiveness of the proposal. For comparative experiments, the channel attention mechanism (CAM) adopted by CBAM is taken as the control method to FMAttn.

In terms of dataset ImageNet100 (Li et al., 2022), it is a subset dataset of the ImageNet Large Scale Visual Recognition Challenge (ILSVRC 2012) for evaluating the performance of DNNs, which are comprised of 100 classifies with 129,026 items which are randomly selected from ILSVRC 2012. For the experiments in the research, ImageNet100 is classified into three parts: the training set, validation set, and test set, with a proportion of 16:4:5.

### Preliminary

The hardware platform for the experiments: Intel(R) Core(TM) i9-10900 CPU@2.80 GHz, 4 × 16 GB DDR4 main memory, and GeForce GTX 3080 Ti with CUDA version 11.4. The software environment is the PyTorch of version 1.9.0+cu102 on Ubuntu 20.04.

For the training settings, the optimizer is the momentum-accelerated stochastic gradient descent (SGD) (Sutskever et al., 2013), with the momentum of 0.9, weight $\_$decay of 0.0001, nesterov. The training epochs are set to 155. It needs to be noted that the cosine annealing schedule is adopted for the learning rate strategy, which is defined as follows in the experiments (Loshchilov & Hutter, 2017).

(7) $$lr(n) = l{r_{min}} + \displaystyle{1 \over 2}(l{r_{max}} - l{r_{min}})(1 + cos\left(\displaystyle{{{T_{cur}}} \over {{T_i}}}\pi \right))$$

where *lr(n)* denotes the learning rate of the ${n^{th}}$ training epoch; $l{r_{max}}$ and $l{r_{min}}$ denote the initial and the minimum learning rate which are set to 0.01 and 1e−5 in this article; ${T_i}$ denotes the number of iterations for the ${i_{th}}$ restart, and ${T_i} \in \left\{ {5,10,20,40,80} \right\}$; ${T_{cur}}$ denotes the account of epochs performed since the ${i_{th}}$ restart. The experimental results consist of two sections: the validation accuracies of training with 155 epochs once and twice. This provides us with a more comprehensive and profound understanding of the performance of the FMAPooling and FMAttn. As the twice training improves the validation accuracy in general which is also proved in the following experiments, the section provides both the experimental results of the once training and the twice training. For the analysis of the experimental results, we focus on the data of the twice training.

### Experiments-VGG

For datasets CIFAR10/100 and ImageNet100, the experiments take two VGG network structures being different in number of layers as well as the input/output sizes. In addition, five strategies are established as follows to conduct comparison experiments in this section:

- VGG_A: the baseline VGG network that without channel-attention mechanism and adopts simply the max pooling layer after each conv-x layer;
- VGG_B: the model that takes the proposed standard FMAPooling module to replace the original max pooling layers of the network;
- VGG_C: the control network especially for VGG_B that replace the avg pooling operation with max pooling to verify the effectiveness of FMAPooling module. For simplicity, it is named as FMMPooling;
- VGG_D: the network that takes both FMAPooling module and FMAttn method, and the FMAttn is added at the end of each conv-x layer;
- VGG_E: the control network especially for VGG_D that takes channel-attention mechanism proposed in CBAM to verify the effectiveness of FMAttn method. And the attention is added as the same with VGG_D.

#### CIFAR10/100

As the standard VGG networks proposed in Simonyan & Zisserman (2015) are heavily overparameterized for CIFAR10/100 with a large number of redundant parts, we adopt a lightweight VGG model with BN opeartions (VGGBN) in the experiments. Table 1 shows the detailed network architecture: only six convolutional layers are deployed and the original three fully-connected layers are reduced to one.

**Table 1:** VGG configuration for CIFAR10/100 (Simonyan & Zisserman, 2015).

Layer	Output size	Network configuration
conv-1	$32 \times 32$	$\left[ {3 \times 3,64} \right] \times 2$
	$16 \times 16$	maxpool
conv-2	$16 \times 16$	$\left[ {3 \times 3,64} \right] \times 2$
	$8 \times 8$	maxpool
conv-3	$8 \times 8$	$\left[ {3 \times 3,128} \right] \times 2$
	$4 \times 4$	maxpool
	2 × 2	AdaptiveAvgPool
	1 × 1	FC-10/100

**Notes:**

$[m \times m,n]\times k$, $m \times m$: convolution kernel size, *n*: output channels; *k*: the repeat number of the layer.

Each convolutional layer is followed by a BN and ReLU layer.

maxpool: [ $2 \times 2$] kernel with stride 2.

Output size: $width \times height$.

Table 2 shows the experimental results of the VGG networks on CIFAR10/100. According to the data of the twice training, conclusions could be drawn as follows.

**Table 2:** Experimental results of VGG on CIFAR10/100.

Strategy	Pooling	Attn.	CIFAR10		CIFAR100	
			Acc.1 (%)	Acc.2 (%)	Acc.1 (%)	Acc.2 (%)
VGG_A	max pooling	N	91.29	91.61	68.49	69.47
VGG_B	FMAPooling	N	92.24	92.31	70.97	71.10
VGG_C	FMMPooling	N	91.76	92.24	70.13	70.93
VGG_D	FMAPooling	FMAttn	**92.82**	**93.17**	**71.59**	**72.29**
VGG_E	FMAPooling	CAM	92.75	93.02	70.47	71.55

**Notes:**

Acc.1: training with 155 epochs once.

Acc.2: training with 155 epochs twice.

The best results are highlighted in bold font.

- In terms of CIFAR10, the accuracy of VGG_B, which utilizes the proposed FMAPooling, is 0.70% higher than that of the baseline model VGG_A, 0.07% higher than that of the control model VGG_C which applies FMMPooling. As for CIFAR100, the accuracy of VGG_B is 1.63% higher than VGG_A, and 0.17% higher than that of VGG_C.
- For CIFAR10, the accuracy of VGG_D, which adopts the proposed FMAttn attention, is 1.56% higher than the baseline model VGG_A, and 0.15% higher than that of the control model VGG_E which takes the channel attention CAM; As for CIFAR100, the accuracy of VGG_D is 2.82% higher than VGG_A, and 0.74% higher than that of the control model VGG_E.

#### ImageNet100

In terms of the network structure of VGG for ImageNet100, eight convolutional layers and one fully-connected layer are applied. The detailed architecture of the network is shown in Table 3.

**Table 3:** VGG configuration for ImageNet100 (Simonyan & Zisserman, 2015).

Layer	Output size	Network configuration
conv-1	$224 \times 224$	$\left[ {3 \times 3,64} \right] \times 2$
	$112 \times 112$	maxpool
conv-2	$112 \times 112$	$\left[ {3 \times 3,64} \right] \times 2$
	$56 \times 56$	maxpool
conv-3	$56 \times 56$	$\left[ {3 \times 3,128} \right] \times 1$
	$28 \times 28$	maxpool
conv-4	$28 \times 28$	$\left[ {3 \times 3,128} \right] \times 1$
	$14 \times 14$	maxpool
conv-5	$14 \times 14$	$\left[ {3 \times 3,128} \right] \times 1$
	$7 \times 7$	maxpool
conv-6	$7 \times 7$	$\left[ {3 \times 3,128} \right] \times 1$
	2 × 2	AdaptiveAvgPool
	1 × 1	FC-100

**Notes:**

$[m \times m,n]\times k$, $m \times m$: convolution kernel size, *n*: output channels; *k*: the repeat number of the layer.

Each convolutional layer is followed by a BN and ReLU layer.

maxpool: $2 \times 2$ with stride 2.

Output size: $width \times height$.

Table 4 shows the experimental results. According to the data of the twice training, conclusions could be drawn as follows:

**Table 4:** Experimental results of VGG on ImageNet100.

Strategy	Pooling	Attn.	ImageNet100	
			Acc.1 (%)	Acc.2 (%)
VGG_A	max pooling	N	71.16	71.67
VGG_B	FMAPooling	N	71.69	72.98
VGG_C	FMMPooling	N	70.82	72.09
VGG_D	FMAPooling	FMAttn	**76.22**	**77.08**
VGG_E	FMAPooling	CAM	74.01	74.98

**Notes:**

Acc.1: training with 155 epochs once.

Acc.2: training with 155 epochs twice.

The best results are highlighted in bold font.

- The accuracy of VGG_B, which utilizes the proposed FMAPooling, is 1.31% higher than that of the baseline model VGG_A, 0.89% higher than that of the control model VGG_C which applies FMMPooling.
- The accuracy of VGG_D, which adopts the proposed FMAttn attention, is 5.06% higher than the baseline model VGG_A, and 2.21% higher than that of the control model VGG_E which takes the channel attention CAM;

### Experiments-ResNet

In terms of experiments concerning ResNet, two networks are applied for datasets CIFAR10/100 and ImageNet100 respectively being difference in number of layers as well as the Input/Output sizes. In detail, three strategies are designed to conduct comparative experiments.

- ResNet_A: the baseline model that without channel-attention mechanism and adopts simply one max pooling layer after the convolutional layer;
- ResNet_B: the network that takes the proposed FMAttn, and three FMAttn modules are added after each convN_x;
- ResNet_C: the control network that takes the channel-attention mechanism proposed in CBAM to verify the effectiveness of FMAttn.

#### ResNet on CIFAR10/100

The detailed networks of ResNet for CIFAR10/100 are shown in Table 5. Being different from the baseline model, the networks of strategies ResNet_B and ResNet_C apply the FMAttn and CAM after each convN_x respectively.

**Table 5:** ResNet configuration for CIFAR10/100 (He et al., 2016).

Layer	Output size	Network configuration
conv1	$32 \times 32$	$3 \times 3$, 64, stride 1
conv2 $\_$x	$32 \times 32$	$\left[ {\matrix{ {3 \times 3,32} \cr {3 \times 3,32} \cr } } \right] \times \,3$
	$32 \times 32$	None/FMAttn/CAM
conv3 $\_$x	$16 \times 16$	$\left[ {\matrix{ {3 \times 3,64} \cr {3 \times 3,64} \cr } } \right] \times \,3$
	$16 \times 16$	None/FMAttn/CAM
conv4 $\_$x	$8 \times 8$	$\left[ {\matrix{ {3 \times 3,128} \cr {3 \times 3,128} \cr } } \right] \times \,3$
	$8 \times 8$	None/FMAttn/CAM
	$4 \times 4$	Adaptive average pooling
	$1 \times 1$	FC-10/100

**Notes:**

The first block of convN $\_$x is followed by a downsample layer, except for conv2 $\_$x.

The first convolutional layer of conv3_x and conv4_x has a stride of 2. The rest are all 1.

Each convolutional layer is followed by a BN layer and ReLU layer.

[ $m \times m,n$]: Convolution kernel size $m \times m$, *n* channels.

Output size: $width \times height$.

According to the experimental results of twice training, as is shown in Table 6: in terms of CIFAR10, the accuracy of ResNet_B, which utilizes the proposed FMAttn, is 0.28% higher than that of the baseline model ResNet_A, and 0.06% higher than that of the ResNet_C which adopts the channel attention mechanism CAM; As for CIFAR100, the accuracy of ResNet_B is 0.63% higher than that of ResNet_A and 0.65% higher than that of ResNet_C.

**Table 6:** Experimental results of ResNet on CIFAR10/100.

Strategy	Attn.	CIFAR10		CIFAR100	
		Acc.1 (%)	Acc.2 (%)	Acc.1 (%)	Acc.2 (%)
ResNet_A	N	93.58	94.08	71.28	72.28
ResNet_B	FMAttn	**93.85**	**94.36**	**71.88**	**72.85**
ResNet_C	CAM	93.53	94.30	71.87	72.2

**Notes:**

Acc.1: training with 155 epochs once.

Acc.2: training with 155 epochs twice.

The best results are highlighted in bold font.

#### ResNet on ImageNet100

The networks of ResNet for ImageNet100 are detailed in Table 7. As Table 8 shows, according to the experimental results of twice training: the accuracy of ResNet_B, which applies the proposed FMAttn, is 2.37% higher than that of the baseline model ResNet_A, and 0.45% higher than that of ResNet_C which applies the channel attention mechanism CAM.

**Table 7:** ResNet configuration for ImageNet100 (He et al., 2016).

Layer	Output size	Network configuration
conv1	$224 \times 224$	$3 \times 3$, 64, stride 1
conv2 $\_$x	$224 \times 224$	$\left[ {\matrix{ {3 \times 3,32} \cr {3 \times 3,32} \cr } } \right] \times \,2$
	$224 \times 224$	None/FMAttn/CAM
conv3 $\_$x	$112 \times 112$	$\left[ {\matrix{ {3 \times 3,64} \cr {3 \times 3,64} \cr } } \right] \times \,2$
	$112 \times 112$	None/FMAttn/CAM
conv4 $\_$x	$56 \times 56$	$\left[ {\matrix{ {3 \times 3,64} \cr {3 \times 3,64} \cr } } \right] \times \,2$
	$56 \times 56$	None/FMAttn/CAM
conv5 $\_$x	$28 \times 28$	$\left[ {\matrix{ {3 \times 3,128} \cr {3 \times 3,128} \cr } } \right] \times \,2$
	$28 \times 28$	None/FMAttn/CAM
conv6 $\_$x	$14 \times 14$	$\left[ {\matrix{ {3 \times 3,128} \cr {3 \times 3,128} \cr } } \right] \times \,2$
	$14 \times 14$	None/FMAttn/CAM
	$4 \times 4$	Adaptive average pooling
	$1 \times 1$	FC-100

**Notes:**

The first block of convN $\_$x is followed by a downsample layer, except for conv2 $\_$x.

The first convolutional layer of conv3_x and conv4_x has a stride of 2. The rest are all 1.

Each convolutional layer is followed by a BN layer and ReLU layer.

[ $m \times m,n$]: Convolution kernel size $m \times m$, n channels.

Output size: $width \times height$.

**Table 8:** Experimental results of ResNet on ImageNet100.

Strategy	Attn.	ImageNet100	
		Acc.1 (%)	Acc.2 (%)
ResNet_A	N	76.42	77.53
ResNet_B	FMAttn	**79.01**	**79.90**
ResNet_C	CAM	78.92	79.45

**Notes:**

Acc.1: training with 155 epochs once.

Acc.2: training with 155 epochs twice.

The best results are highlighted in bold font.

### Experiments-MobileNetV2

**Figure 5:**
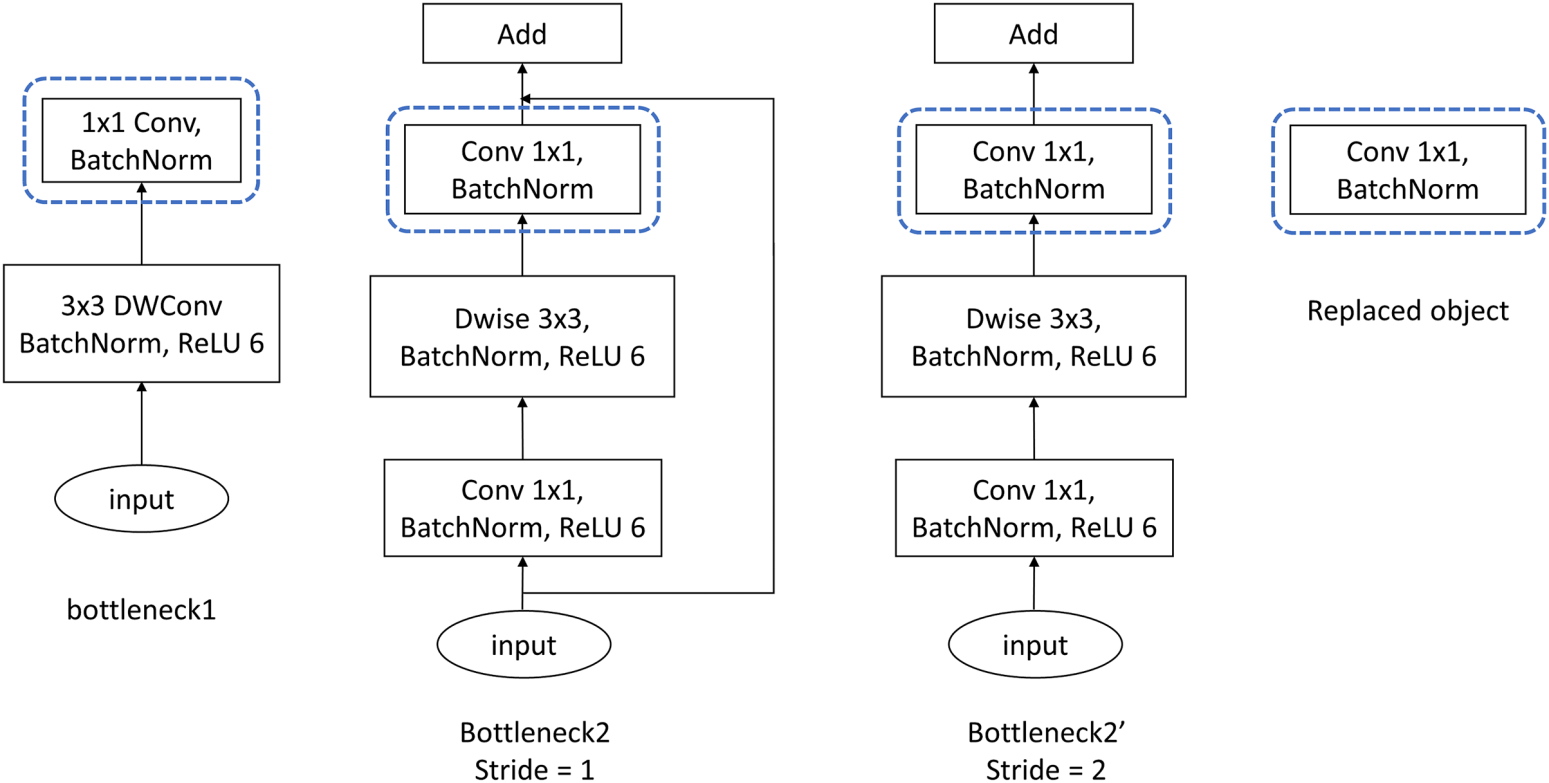
Bottleneck structure of MobileNetV2. Dwise, Depthwise convolution.

For MobileNetV2, the experiments are conducted simply on ImageNet100. Being different from experimental strategies of VGG and ResNet, the proposed FMAttn is applied for MobileNetV2 by replacing the last [ $1 \times 1$] convolutional layer within the MobileNetV2-Bottlenecks rather than directly added to the network, as is shown in Fig. 5. In detail, the experimental strategies are established as follows:

- MobilenetV2_A: the baseline model which is the standard MobileNetV2 as is proposed in Sandler et al. (2018);
- MobilenetV2_B: the network with the last [ $1 \times 1$] convolutional layer of the MobileNetV2-Bottlenecks replaced by the proposed FMAttn;
- MobilenetV2_C: the control network that utilizes the channel-attention mechanism proposed in CBAM to replace the last [ $1 \times 1$] convolutional layer of the MobileNetV2-Bottlenecks.

Table 9 shows the experimental results of MobileNetV2 on ImageNet100: based on the analysis of the twice training, the accuracy of MobileNetV2_B is 0.53% higher than that of the baseline model MobileNetV2_A, and 1.80% higher than that of the control model MobileNetV2_C which takes the channel attention mechanism CAM.

**Table 9:** Experimental results of MobileNetV2 on ImageNet100.

Strategy	Attn.	ImageNet100	
		Acc.1 (%)	Acc.2 (%)
MobileNetV2_A	N	76.5	76.5
MobileNetV2_B	FMAttn	**77.03**	**77.03**
MobileNetV2_C	CAM	75.15	75.23

**Notes:**

Acc.1: training with 155 epochs once.

Acc.2: training with 155 epochs twice.

### Summary

According to the experimental results and analysis above, conclusions could be drawn as follows:

- In terms of VGG networks on datasets CIFAR10/100 and ImageNet100, the FMAPooling improves the validation by 1.31–1.63% compared with the baseline; the FMAttn together with the FMAPooling improves the performance by 1.56–5.06% compared with the baseline and 0.15–2.21% compared with the channel attention mechanism CAM.
- For ResNet models on datasets CIFAR10/100 and ImageNet100, the FMAttn improves the performance by 0.28–2.37% compared with the baseline and 0.06–0.65% compared with the channel attention mechanism CAM.
- As for MobileNetV2 on dataset ImageNet100, the FMAttn improves the performance by 0.53% compared with the baseline and 1.80% compared with the channel attention mechanism CAM.

In summary, the experiments by conducting VGG, ResNet, and MobileNetV2 on datasets CIFAR10/100 as well as ImageNet100 prove the effectiveness of the proposed FMAPooling and FMAttn. Although the improvement in the DNNs’ performance varies with DNN architectures as well as datasets, the superiority of FMAttn to the previous studies on the channel attention mechanism is verified. As for the application, the proposed FMAttn could be directly integrated into various DNN architectures, as is shown in the experiments of VGG and ResNet; Furthermore, the FMAttn could be also conveniently applied by replacing certain layers of DNNs for utilizing the DNN architectures such as saving computation overhead, as is shown in the experiments of MobileNetV2.

## Conclusion

The representation ability of feature maps is crucial for DNNs. Numerous studies have focused on improving the efficiency of feature maps. In this article, we propose the FMAPooling function and an improved channel attention mechanism FMAttn to enhance the representation of feature maps. The proposals could be directly integrated into various DNN networks. In addition, to optimize the architectures of DNNs, the proposals could also conveniently take the place of certain structures of DNNs. The effectiveness of the proposals is proven with sufficient experimental data: FMAPooling improves the performance by up to 1.63% compared with the baseline. FMAttn improves the performance by up to 2.21% compared with the channel attention mechanism adopted in CBAM. The pooling functions, as well as the self-attention mechanism, provide promising solutions for enhancing feature maps. For future work, we plan to make in more depth investigation of the underlying principles of the pooling function with the self-attention mechanisms, for the purposes of designing more effective DNN architectures.

## Additional Information and Declarations

### Data Availability

The following information was supplied regarding data availability: The codes are available at GitHub: https://github.com/lihengyi-ai/Enhanced_Mechanisms. The raw data of the experimental results are available at GitHub: https://github.com/lihengyi-ai/Enhanced_Mechanisms, and at Zenodo: lihengyi-ai. (2022). lihengyi-ai/Enhanced_Mechanisms: Enhanced Mechanisms of Pooling and Channel-Attention for Deep Learning Feature Maps (Version V2). Zenodo. https://doi.org/10.5281/zenodo.7127664.

## References

[ref-1] Chen K, Yao L, Zhang D, Wang X, Chang X, Nie F (2020). A semisupervised recurrent convolutional attention model for human activity recognition. IEEE Transactions on Neural Networks and Learning Systems.

[ref-2] Dong Y, Liu Y, Kang H, Liu P, Liu Z (2022). Lightweight and efficient neural network with SPSA attention for wheat ear detection. PeerJ Computer Science.

[ref-3] Duan K, Bai S, Xie L, Qi H, Huang Q, Tian Q (2019). CenterNet: keypoint triplets for object detection.

[ref-4] Fujikawa Y, Li H, Yue X, Aravinda CV, Prabhu GA, Meng L (2022). Recognition of oracle bone inscriptions by using two deep learning models. International Journal of Digital Humanities.

[ref-5] He K, Zhang X, Ren S, Sun J (2015). Spatial pyramid pooling in deep convolutional networks for visual recognition. IEEE Transactions on Pattern Analysis and Machine Intelligence.

[ref-6] He K, Zhang X, Ren S, Sun J (2016). Deep residual learning for image recognition.

[ref-7] Howard AG, Zhu M, Chen B, Kalenichenko D, Wang W, Weyand T, Andreetto M, Adam H (2017). MobileNets: efficient convolutional neural networks for mobile vision applications. CoRR.

[ref-8] Hu J, Shen L, Sun G (2018). Squeeze-and-excitation networks.

[ref-9] Huang G, Liu Z, Maaten LVD, Weinberger KQ (2017). Densely connected convolutional networks.

[ref-10] Ian G, Yoshua B, Aaron C (2016). Deep learning.

[ref-11] Law H, Deng J (2020). CornerNet: detecting objects as paired keypoints. International Journal of Computer Vision.

[ref-12] Li H, Wang Z, Yue X, Wang W, Hiroyuki T, Meng L (2021). A comprehensive analysis of low-impact computations in deep learning workloads.

[ref-13] Li H, Yue X, Wang Z, Chai Z, Wang W, Tomiyama H, Meng L (2022). Optimizing the deep neural networks by layer-wise refined pruning and the acceleration on FPGA. Computational Intelligence and Neuroscience.

[ref-14] Li Q, Zhang A, Liu P, Li J, Li C (2020). A novel CSI feedback approach for massive MIMO using LSTM-attention CNN. IEEE Access.

[ref-15] Lin M, Chen Q, Yan S (2014). Network in network.

[ref-16] Liu S, Deng W (2015). Very deep convolutional neural network based image classification using small training sample size.

[ref-17] Liu P, Huda MN, Sun L, Yu H (2020). A survey on underactuated robotic systems: bio-inspiration, trajectory planning and control. Mechatronics.

[ref-18] Liu P, Yu H, Cang S (2018). Geometric analysis-based trajectory planning and control for underactuated capsule systems with viscoelastic property. Transactions of the Institute of Measurement and Control.

[ref-19] Liu P, Yu H, Cang S (2019). Adaptive neural network tracking control for underactuated systems with matched and mismatched disturbances. Nonlinear Dynamics.

[ref-20] Loshchilov I, Hutter F (2017). SGDR: stochastic gradient descent with warm restarts.

[ref-21] Saho K, Hayashi S, Tsuyama M, Meng L, Masugi M (2022). Machine learning-based classification of human behaviors and falls in restroom via dual doppler radar measurements. Sensors.

[ref-22] Sandler M, Howard A, Zhu M, Zhmoginov A, Chen L-C (2018). MobileNetV2: Inverted residuals and linear bottlenecks.

[ref-23] Simonyan K, Zisserman A (2015). Very deep convolutional networks for large-scale image recognition.

[ref-24] Sutskever I, Martens J, Dahl G, Hinton G (2013). On the importance of initialization and momentum in deep learning.

[ref-25] Szegedy C, Liu W, Jia Y, Sermanet P, Reed S, Anguelov D, Erhan D, Vanhoucke V, Rabinovich A (2015). Going deeper with convolutions.

[ref-26] Vaswani A, Shazeer N, Parmar N, Uszkoreit J, Jones L, Gomez AN, Kaiser L, Polosukhin I (2017). Attention is all you need.

[ref-27] Wang Q, Wu B, Zhu P, Li P, Zuo W, Hu Q (2020). ECA-Net: efficient channel attention for deep convolutional neural networks.

[ref-28] Woo S, Park J, Lee J-Y, Kweon IS (2018). CBAM: convolutional block attention module.

[ref-29] Yu D, Wang H, Chen P, Wei Z (2014).

[ref-30] Yue X, Li H, Fujikawa Y, Meng L (2022a). Dynamic dataset augmentation for deep learning-based oracle bone inscriptions recognition. ACM Journal on Computing and Cultural Heritage.

[ref-31] Yue X, Li H, Shimizu M, Kawamura S, Meng L (2022b). YOLO-GD: a deep learning-based object detection algorithm for empty-dish recycling robots. Machines.

[ref-32] Zhang L, Schaeffer H (2019). Forward stability of ResNet and its variants. Journal of Mathematical Imaging and Vision.

[ref-33] Zhang X, Zhou X, Lin M, Sun J (2018). ShuffleNet: an extremely efficient convolutional neural network for mobile devices.

[ref-34] Zhou, Chellappa (1988). Computation of optical flow using a neural network.

